# Improve word embedding using both writing and pronunciation

**DOI:** 10.1371/journal.pone.0208785

**Published:** 2018-12-10

**Authors:** Wenhao Zhu, Xin Jin, Jianyue Ni, Baogang Wei, Zhiguo Lu

**Affiliations:** 1 School of Computer Engineering and Science, Shanghai University, Shanghai, China; 2 College of Computer Science and Technology, Zhejiang University, Zhejiang, China; 3 Library of Shanghai University, Shanghai University, Shanghai, China; The University of Memphis, UNITED STATES

## Abstract

Text representation can map text into a vector space for subsequent use in numerical calculations and processing tasks. Word embedding is an important component of text representation. Most existing word embedding models focus on writing and utilize context, weight, dependency, morphology, etc., to optimize the training. However, from the linguistic point of view, spoken language is a more direct expression of semantics; writing has meaning only as a recording of spoken language. Therefore, this paper proposes the concept of a pronunciation-enhanced word embedding model (PWE) that integrates speech information into training to fully apply the roles of both speech and writing to meaning. This paper uses the Chinese language, English language and Spanish language as examples and presents several models that integrate word pronunciation characteristics into word embedding. Word similarity and text classification experiments show that the PWE outperforms the baseline model that does not include speech information. Language is a storehouse of sound-images; therefore, the PWE can be applied to most languages.

## Introduction

Word representation plays a very important role in natural language processing (NLP). The key issue is how to obtain word semantics. The initial word representation, which simply digitizes the word, cannot capture its semantics. Subsequent models that can capture semantics benefit from the distributional hypothesis of Harris [1], which assumes that words with similar contexts have similar meanings. The most efficient word embedding models are based on this concept.

Neural networks are important machine learning models that show superiority in many areas. The application of neural networks to word embedding models was proposed by Bengio et al. as early as 2003 [2]. Since then, neural network language modeling has gained attention in word representation and is now a popular technique [3–5]. However, the computation involved in neural networks is intensive. Mikolov et al. [6] proposed the continuous bag-of-words model (CBOW) and the Skip-gram model, which are highly efficient and can be trained on large-scale corpora.

To improve the quality of word embedding, researchers have focused on morphology, which refers to the elements that compose a word, such as prefixes and suffixes in English or the characters that make up a word in Chinese. Various researchers [7–9] have incorporated morphology into word embedding training. As such, word embedding models have been developed and have shown superior performance in a variety of NLP tasks, including dialog systems [10], sentiment analyses [11], machine translation [12], and text classification [13].

However, most existing models have focused on the writing itself, ignoring the fact that spoken language expresses the meaning directly, whereas writing is simply a way to record speech. One of the basic principles of modern linguistic theory considers that only spoken words truly reflect the concepts and that writing is simply a record of the spoken language, analogous to a phonograph [14, 15].

In speech processing systems, Bengio and Heigold utilize speech signal directly [16]. They project the signal and words into an embedding space where words that sound alike are nearby in the Euclidean sense. Kamper [17] and He [18] conducted a deeper study based on this. Levin [19] applied acoustic segment embedding to zero resource query-by-example keyword search. These acoustic embeddings which mainly capture phonetic structure are different from word embeddings.

Based on this, this paper proposes the pronunciation-enhanced word embedding model (PWE), which incorporates speech information into the model. Pinyin and phonetic symbols are both direct descriptions of word pronunciation. They both capture aspects of speech not present in the writing system. Therefore, this paper incorporates speech information through adding phonetic symbols or pinyin into the model. The PWE is highly scalable from two perspectives. First, the PWE can easily be combined with existing models. Second, the PWE can be applied to most languages.

This paper presents several methods for combining words and speech information for the Chinese language, English language and Spanish language to construct the PWE. The PWE outperforms a baseline model in word similarity and text classification experiments. In addition, this paper finds that the pronunciation embedding captures semantics and that word embedding contains speech information by revealing the semantic correlation between word embedding and pronunciation embedding. This paper confirms that including pronunciation improves the quality of word embedding.

## Related work

### Word2vec and related models

Word2vec is an efficient word embedding model that uses a neural network, as proposed by Mikolov et al. [6], and includes the CBOW and the Skip-gram model. The CBOW predicts a target word from its context, and the Skip-gram model uses a word to predict its context. Context is acquired by a sliding window. Given a word sequence D = {*x*_1_,*x*_2_,…,*x*_*N*_}, the CBOW maximizes the following average log probability:

$$L=\frac{1}{N}\sum_{i=1}^{N}log\;p(x_{i}|x_{i-j},x_{i-j+1},\ldots ,x_{i+j}).$$ (1)

whereas the objective of the Skip-gram model is denoted by

$$L=\frac{1}{N}\sum_{i=1}^{N}\sum_{-j\leq c\leq j,c\neq 0}log\;p(x_{i+c}|x_{i}).$$ (2)

Here, *j* is the context window size. Word2vec uses the following softmax function to calculate the probability:

$$p(x_{O}|x_{I})=\frac{exp({v_{x_{O}}^{\prime }}^{T}*v_{x_{I}})}{\sum_{x\in W}exp({v_{x}^{\prime }}^{T}*v_{x_{I}})},$$

where *W* denotes the dictionary; and *v*_x_ and $v_{x}^{\prime }$ are the input and output word embeddings of word *x*, respectively. A large-scale corpus exists; therefore, hierarchical softmax and negative sampling are used to improve training efficiency [20].

Subsequently, many models have been proposed to improve word2vec. Le and Mikolov [21] modified word2vec to represent sentences and documents. Qiu et al. [22] considered proximity and ambiguity to improve word2vec. Levy and Goldberg [23] generalized the Skip-gram model moving the focus from linear bag-of-words contexts to arbitrary word contexts.

### Improvement from morphology

Several researchers have added morphology to word embedding models to improve the quality of word embedding. Botha and Blunsom [7] assumed that word vectors comprise a linear function of arbitrary sub-elements of the word, e.g., surface form, stem, affixes, and other latent information. For example, the word “unbelievable” consists of “un”, “believ” and “able.” Xu and Liu [8] observe that morphemes have meaning. For example, words ending with the suffix “able” carry the meaning of “capable.” Therefore, morphemes are replaced by their meanings in the model.

For languages such as Chinese, the characters in a word also contain rich semantics. In Chinese, the meaning of the word “教室” (classroom) can be extracted from its two characters, “教” (teach) and “室” (room). Many similar words exist in Chinese. Therefore, Chen et al. [9] proposed a character-enhanced word embedding model (CWE) that integrates characters into training to jointly learn the characters and word embedding. Suppose that the character sequence of word *x*_*t*_ is {*c*_1_,*c*_2_,…,*c*_*k*_}, where *c*_*k*_ denotes the *kth* character of *x*_*t*_. The modified word embedding is defined as

$$v_{\hat{x_{t}}}=v_{x_{t}}+\frac{1}{k}\sum_{j=1}^{k}v_{c_{j}},$$

where $v_{x_{t}}$ is the original word embedding, *k* is the number of characters, and $v_{c_{j}}$ represents the *jth* character. In addition, the CWE proposes several methods to solve character ambiguities, including position-based, cluster-based and nonparametric cluster-based character embedding. The similarity-based character-enhanced word embedding(SCWE) improves the CWE by including the semantic contributions of characters to a word [24].

## Writing is symbolized language

Linguists have long discussed the relationship between writing and language. At the beginning of the 20^th^ century, Saussure [14] proposed that language is a storehouse of sound-images and that writing is a tangible form of those images. Language is primarily an auditory symbol system, whereas the written forms of language are secondary symbols (symbols of symbols) that represent the spoken symbols [25]. For a linguist, writing is, except for certain matters of detail, merely an external preservation device, similar to a phonograph, which stores observations about features of historical speech [15]. Therefore, writing is a symbolization of the language symbols that are the basic principles of linguistics.

The Chinese, English and Spanish languages are three different kinds of language in terms of the relationship between words and their pronunciations. In Chinese language, there is no connection between the word and its pronunciation. In English, due to the complex pronunciation rules, we can only guess the pronunciation from the spelling of a word. In Spanish, almost every character has a fixed pronunciation, so we can obtain the pronunciation of a word directly. Therefore, this paper uses the Chinese, English and Spanish languages as examples to create the PWE. In addition, pinyin is used to represent the pronunciation of Chinese characters. For an introduction to Pinyin, please refer to https://en.wikipedia.org/wiki/Pinyin.

## The PWE

### The basic model

The PWE reflects the linguistic theory of the relationship between writing and language; the model considers that spoken language is the direct expression of semantics and that writing is a record of spoken language. Therefore, the PWE integrates word pronunciations into the word embedding model. The key addition is the PWE's integration of pronunciation into word embedding. Suppose *p* is the pronunciation of word *w*, vector *v*_*w*_ represents the word *w*, and vector *v*_*p*_ represents the pronunciation *p*. Then, the modified word embedding to include the pronunciation is defined as follows:

$$\hat{v_{w}}=v_{w}+v_{p}.$$

This is the basic idea for obtaining a modified word embedding, and this idea can change according to specific circumstances. After acquiring $\hat{v_{w}}$, other existing word embedding models can be used to train pronunciation and word embedding. In the following sections, this paper introduces concrete PWEs based on specific word embedding models.

### The PWE based on word2vec

Word2vec includes 2 models. This section uses the CBOW as an example to introduce the PWE based on word2vec.

**Fig 1:**
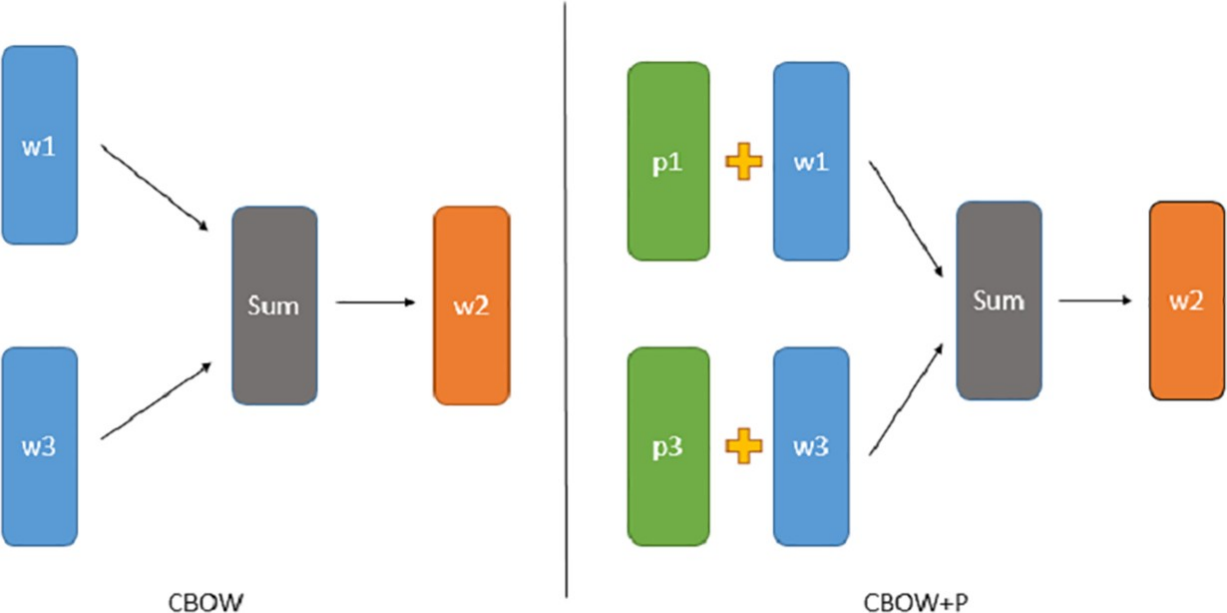
The CBOW and the CBOW+P. The structure of the CBOW and the CBOW+P given a word sequence (w1,w2,w3} in which word w2 is predicted by word w1 and w3. In the CBOW+P, p1 and p3 are the pronunciations of words w1 and w3, respectively.

The CBOW and PWE based on the CBOW (denoted as CBOW+P) are shown in Fig 1. The difference between the CBOW+P and the CBOW lies in the different methods used to construct the word embedding. The CBOW+P adds pronunciation embedding to the word embedding. Both models predict a target word from context, and the CBOW+P does not attempt to predict the target pronunciation. Therefore, the CBOW+P and the CBOW share the same objective as shown previously in Eq (1).

Let *W* denote the dictionary and *P* denote the pronunciation set. A word *w*_*i*_∈*W* is represented by the word embedding $v_{w_{i}}$, and its pronunciation *p*_*i*_∈*P* is represented by the pronunciation embedding $v_{p_{i}}$. Assume that *p*_*t*_ is the pronunciation of word *w*_*t*_. Then, the word embedding of the CBOW+P is defined as,

$$v_{\hat{w_{t}}}=v_{w_{t}}+v_{p_{t}}$$ (3)

After acquiring the modified word embedding, the CBOW+P can be trained similarly to the CBOW; however, the CBOW+P jointly learns both the word embedding and the pronunciation embedding. This study used hierarchical softmax to improve the training efficiency.

The PWE based on the Skip-gram model (denoted as the Skip-Gram+P) also uses Eq (3) to modify the word embedding, and its objective is Eq (2).

### The PWE based on the CWE

The CWE assumes that in languages such as Chinese, a word is usually composed of several characters and contains rich internal information [9]. Therefore, the CWE integrates character information into the word embedding model. This section describes a basic construction method for the PWE based on the CWE, and the following section presents another construction method for the Chinese language example.

In this basic method, word embedding consists of three parts: the word, the word's characters and the word's pronunciation. Let *W* denote the dictionary, *C* denote the set of characters and *P* denote the set of pronunciations. Suppose that the character sequence of word *w*_*t*_∈*W* is D = {*c*_1_,*c*_2_,…,*c*_*k*_}, where *c*_*i*_∈*C* and *p*_*t*_∈*P* is the pronunciation of word *w*_*t*_. $v_{\hat{w_{t}}}$ is then used to represent the modified word embedding:

$$v_{\hat{w_{t}}}=v_{w_{t}}+\frac{1}{k}\sum_{j=1}^{k}v_{c_{j}}+v_{p_{t}}.$$

Here, $v_{w_{t}}$ is the original word embedding, $v_{c_{j}}$ is the embedding that corresponds to the *jth* character in the word, and $v_{p_{t}}$ is the embedding that corresponds to the *p*_*t*_.

After acquiring the modified word embedding, the PWE based on the CWE can be trained similarly to the CWE; however, the PWE based on the CWE jointly learns the word embedding, the character embedding and the pronunciation embedding.

### The PWE in different languages

This paper implemented the PWE based on word2vec for Chinese, English and Spanish. In addition, this study implemented the PWE based on the CWE for Chinese.

For word2vec, in Chinese, let *P* denote the pinyin set, and pinyin *p*_*j*_∈*P* is represented by embedding $v_{p_{j}}$. Let *W* denote the dictionary, where word *w*_*t*_∈*W* is represented by the word embedding $v_{w_{t}}$. Suppose word *w*_*t*_ includes *k* characters, and the corresponding pinyin sequence is D = {*p*_1_,*p*_2_,…,*p*_*k*_}. The modified word embedding that adds the pronunciation is

$$v_{\hat{w_{t}}}=v_{w_{t}}+\frac{1}{k}\sum_{j=1}^{k}v_{p_{j}}.$$

For English and Spanish, suppose *p*_*t*_ is the pronunciation of word *w*_*t*_; the modified word embedding is

$$v_{\hat{w_{t}}}=v_{w_{t}}+v_{p_{t}}$$

This paper proposes two methods for merging the PWE based on the CWE for the Chinese language. The first method is to directly add the pronunciation vector based on the CWE, as described in the previous section, denoted as the CWE+P1. The modified word embedding consists of word, character and pronunciation embeddings. Assume that the character sequence of word *w*_*t*_∈*W* is D = {*c*_1_,*c*_2_,…,*c*_*k*_}, and its corresponding pinyin sequence is H = {*p*_1_,*p*_2_,…,*p*_*k*_}, where *p*_*i*_ is the pinyin of character *c*_*i*_. The modified word embedding is defined as follows:

$$v_{\hat{w_{t}}}=v_{w_{t}}+\frac{1}{k}\sum_{j=1}^{k}(v_{c_{j}}+v_{p_{j}}),$$

where $v_{w_{t}}$ is the original word embedding; and $v_{c_{j}}$ and $v_{p_{j}}$ are the embedding of character *c*_*j*_ and pinyin *p*_*j*_, respectively. For example, the word “教室” (classroom) includes two characters, “教” (teach) and “室” (room). The pinyin of “教” (teach) is “jiao4” and the pinyin of “室” (room) is “shi4.” The modified word embedding is

$$v_{\hat{教室}}=v_{教室}+\frac{1}{2}(v_{教}+v_{jiao4}+v_{室}+v_{shi4})$$

The second method creates an embedding for each pinyin of the character. Based on the specific pronunciation of the character in the word, this method adds the corresponding embedding to the word embedding to achieve the modified word embedding. This method is denoted as the CWE+P2. As described in Section 3, character *c*_*i*_ may have *n* different pronunciations {*p*_1_,*p*_2_,…,*p*_*n*_}; therefore, the CWE+P2 creates *n* embedding values that correspond to those *n* pronunciations. For example, suppose that the character sequence of word *w*_*t*_ is D = {*c*_1_,*c*_2_,…,*c*_*k*_} and the corresponding pinyin sequence is H = {*p*_1_,*p*_2_,…,*p*_*k*_}, where *p*_*i*_ is the pinyin of character *c*_*i*_ in the word *w*_*t*_. The modified word embedding is defined as follows:

$$v_{\hat{w_{t}}}=v_{w_{t}}+\frac{1}{k}\sum_{j=1}^{k}v_{{cp}_{j}}.$$

The embedding $v_{{cp}_{j}}$ corresponds to the pinyin of the *jth* character in the word. For the word “教室” (classroom), embeddings for the pinyin “jiao4” of character “教” (teach) and the pinyin “shi4” of character “室” (room) are created and denoted as $v_{{cp}_{1}}$ and $v_{{cp}_{2}}$, respectively. The modified word embedding for the word “教室” (classroom) is defined as follows:

$$v_{\hat{教室}}=v_{教室}+\frac{1}{2}(v_{{cp}_{1}}+v_{{cp}_{2}}).$$

## Experiments

### Datasets and tools

Using the Chinese, English and Spanish languages as examples, this study conducted an experiment to evaluate the model, selecting the *Chinese Wikipedia Dump*, *English Wikipedia Dump* and *Spanish Wikipedia Dump* corpora to train the model. You can get corpus from http://download.wikipedia.com/zhwiki/, http://download.wikipedia.com/enwiki/ and http://download.wikipedia.com/eswiki/ respectively. For Chinese, word segmentation is necessary; *ANSJ* was used as the word segmentation tool. You can get ANSJ from https://github.com/NLPchina/ansj_seg. ANSJ supports Chinese name recognition and includes a user-defined dictionary. ANSJ can process approximately one million words per second and can reach a word segmentation accuracy greater than 96%. This paper uses *HanLP(refer to* http://hanlp.linrunsoft.com/*)* to convert Chinese words into pinyin. HanLP is a comprehensive Chinese NLP processing tool implemented with Java. HanLP's pinyin conversion tool recognizes polyphones at millisecond response speeds. The context window size is set to 5, and the embedding dimension sizes are set to 100.

### Word similarity

In the experiment, the cosine similarity of word embedding is used to indicate semantic relatedness of a given word pair. A word similarity experiment is implemented to evaluate the quality of word embedding by comparing the semantic relatedness computed by models with human judgments. For this paper, wordsim-240 and wordsim-296 [26] were used as the evaluation datasets for Chinese; for English, MTurk-771 [27], MEN [28], WS-353-SIM and WS-353-REL [29] were used; for Spanish, WS-353 [30] and RG-65 [31] were used. The numbers of word pairs in these datasets are shown in Table 1.

**Table 1:** The number of word pairs in the datasets.

Language	Dataset	Number
**Chinese**	**Wordsim-240**	240
	**Wordsim-296**	296
**English**	**MTurk-771**	771
	**MEN**	3000
	**WS-353-SIM**	203
	**WS-353-REL**	252
**Spanish**	**WS-353**	353
	**RG-65**	65

This paper uses the Spearman correlation ρ to evaluate the relatedness between the model results and human judgment; then, the model's performance on the word similarity experiment is evaluated according to ρ. In this experiment, the word pairs that contain new words are ignored. Each model is trained a minimum of 10 times; consequently, the experiment acquired at least 10 different results for each model. Tables 2–4 show the averaged experimental results for the different languages.

**Table 2:** Experimental results of word similarity (ρ * 100) for Chinese.

	model	wordsim-240	wordsim-296
**CBOW Based**	**CBOW**	54.39	61.32
	**CBOW+P**	**56.15**	**64.20**
	**CWE**	55.14	63.33
	**CWE+P1**	55.63	63.62
	**CWE+P2**	54.9	63.18
**Skip-gram Based**	**Skip-gram**	**58.85**	62.89
	**Skip-gram+P**	58.53	62.62
	**CWE**	58.60	63.88
	**Cwe+p1**	58.75	63.64
	**CWE+P2**	58.49	**63.97**

**Table 3:** Experimental results of word similarity (ρ * 100) for English.

	model	MTurk-771	WS-353-REL	Men	WS-353-SIM
**CBOW Based**	**CBOW**	55.14	59.23	62.34	64.59
	**CBOW+P**	**55.82**	**59.75**	**63.44**	**65.39**
**Skip-Gram Based**	**Skip-Gram**	56.61	59.17	**67.62**	62.04
	**Skip-Gram+P**	**56.67**	**60.03**	67.44	**62.70**

**Table 4:** Experimental results of word similarity (ρ * 100) for Spanish.

	model	WS-353	RG-65
**CBOW Based**	**CBOW**	20.81	61.07
	**CBOW+P**	**22.16**	**65.25**
**Skip-Gram Based**	**Skip-Gram**	**17.80**	66.37
	**Skip-Gram+P**	17.02	**69.42**

From the experimental results of the model based on the CBOW, a number of results can be observed. Regardless of languages and datasets, the best results are obtained by the CBOW+P. The CBOW+P outperforms the CBOW for all the datasets and languages. For Chinese, the ρ of the CBOW+P increases by 4.7% on wordsim-296. For Spanish, the ρ of the CBOW+P increases by 6.8% on RG-65. The models that include pronunciation information generally performed better than the benchmark models. For Chinese, the CBOW+P and the CWE+P1 outperformed the corresponding benchmark models. For English and Spanish, the CBOW+P outperformed the CBOW.

From the experimental results of the model based on the Skip-gram model, we observe that the PWE based on the Skip-gram model achieves good results. For English, the ρ of the Skip-gram+P model is better than that of the Skip-gram model on MTurk-771, WS-353-SIM and WS-353-REL. For Spanish, the ρ of the Skip-gram+P model increases by 4.6% on RG-65. However, the experimental results based on the Skip-gram model are somewhat weaker than the experimental results based on the CBOW. This suggests that it is difficult to predict surrounding sounds based on only one sound, but given a sound sequence that lacks a sound, it is easy to guess the missing sound based on the surrounding sounds, which likely explains why the experimental results based on the CBOW are better than those based on the Skip-gram model. From this experiment, we can observe that the PWE performs well for different languages.

### Text classification

For text classification experiments, this paper adopted the *Fudan* (refer to http://www.datatang.com/data/44139), *Sogou* (refer to http://download.labs.sogou.com/) and *Netease* (refer to http://www.datatang.com/data/1196) corpora for Chinese, 20Newsgroups (refer to http://qwone.com/~jason/20Newsgroups/) for English and TASS 2017 [32] for Spanish as the experimental datasets. The Fudan corpus contains 20 categories. The number of documents in each category ranges from tens to thousands. For the experiment presented in this paper, 5 categories were selected, each of which contains more than 1,000 documents. Table 5 shows the categories and the corresponding numbers of documents. The documents include various types of papers and news reports. Sogou and Netease are news classification datasets. Sogou includes nine categories; each category includes 1,990 documents. Netease includes six categories with 4,000 documents each. 20Newsgroups contains 20 newsgroups and some of the newsgroups are very closely related to each other. This paper extracted six categories followed by its home page shown in Table 6. The category column indicates the categories that were extracted from the specific categories and the sub-categories column indicates categories that were included in extracted category. TASS 2017 is a sentiment classification dataset that is based on Twitter. TASS 2017 includes “P”, “N”, “NEU” and “NONE” 4 categories and “P”, “N” have the most samples. As such, this paper chose “P” and “N” for the experiment.

**Table 5:** Categories and their sizes selected from the Fudan corpus.

Category	Size
**Environment**	1,218
**Agriculture**	1,022
**Economy**	1,601
**Politics**	1,026
**Sports**	1,254

**Table 6:** Categories of 20Newsgroups.

Category	Sub-categories
**comp**	comp.graphics, comp.os.ms-windows.misc, comp.sys.ibm.pc.hardware, comp.sys.mac.hardware, comp.windows.x
**misc**	misc.forsale
**politics**	talk.politics.misc, talk.politics.guns, talk.politics.mideast
**rec**	rec.autos, rec.motorcycles, rec.sport.baseball, rec.sport.hockey
**religion**	talk.religion.misc, alt.atheism, soc.religion.christian
**sci**	sci.crypt, sci.electronics, sci.med, sci.space

This experiment used the average word embedding in the document to represent the document. The text classifier was trained with LIBLINEAR [33]. For a corpus that does not distinguish between the training set and test set, we used 5-fold cross validation. The accuracies of the models on different languages are shown in Table 7 and their F-measure scores for each category of different corpus are shown in Tables 8–12 respectively. The F-score is a metric that considers both precision and recall.

**Table 7:** Accuracies of models on different corpus.

	Model	Fudan	Sogou	Netease	20Newsgroups	TASS2017
**CBOW Based**	**CBOW**	91.04	82.72	93.1	72.26	66.95
	**CBOW+P**	91.52	82.89	93.52	**72.85**	**68.77**
	**CWE**	91.81	83.24	93.61	-	-
	**CWE+P1**	**91.91**	83.17	**93.67**	-	-
	**CWE+P2**	91.86	**83.38**	93.59	-	-
**Skip-gram Based**	**Skip-gram**	91.91	**84.09**	**94.13**	76.17	**67.91**
	**Skip-gram+P**	91.99	84.03	93.97	**76.65**	67.18
	**CWE**	92.06	84.06	94.04	-	-
	**CWE+P1**	**92.14**	83.96	94.04	-	-
	**CWE+P2**	92.09	83.93	94.07	-	-

**Table 8:** F-score (%) on each category from the Fudan corpus.

	Model	Environment	Agriculture	Economy	Politics	Sports
**CBOW Based**	**CBOW**	92.43	85.2	89.7	92.58	94.83
	**CBOW+P**	93.36	86.48	89.81	92.4	95.29
	**CWE**	93.75	86.86	**90.05**	92.73	95.44
	**CWE+P1**	93.95	**87.15**	90.03	92.8	**95.48**
	**CWE+P2**	**94.13**	86.67	89.92	**92.92**	95.47
**Skip-gram Based**	**Skip-gram**	94.47	87.38	89.78	**92.17**	95.61
	**Skip-gram+P**	94.74	88	89.68	92.01	95.5
	**CWE**	94.62	**88.44**	**89.91**	92	95.29
	**CWE+P1**	**94.86**	88.3	89.9	91.86	**95.67**
	**CWE+P2**	94.77	88.34	89.86	92.03	95.42

**Table 9:** F-score (%) on selected categories from the Sogou corpus.

	Model	Internet	Sports	Healthy	Job	Economy	Education	Culture	Travel	Military
**CBOW** **Based**	**CBOW**	78.35	96.91	82.41	90.39	75.91	80.03	71.5	84.91	84.41
	**CBOW+P**	79.43	97.04	82.61	90.52	74.98	79.83	71.91	85.03	85.11
	**CWE**	79.73	97.18	**83.55**	90.59	75.71	80.14	72.07	85.34	85.31
	**CWE+P1**	79.28	97.06	83.51	**91.11**	74.76	80.02	**72.24**	**85.47**	85.44
	**CWE+P2**	**79.94**	**97.41**	83.33	90.63	**76.08**	**80.4**	72	85.31	**85.72**
**Skip-gram Based**	**Skip-gram**	79.82	97.45	83.93	91.86	77.08	**81.57**	72.91	86.38	86.12
	**Skip-gram+P**	79.98	97.27	84.07	91.59	77.25	81.49	72.51	**86.82**	85.7
	**CWE**	**80.13**	**97.46**	83.85	**91.89**	77.16	81.41	**72.55**	86.29	**86.13**
	**CWE+P1**	79.72	97.43	84	91.46	**77.43**	81.32	72.51	86.48	85.69
	**CWE+P2**	79.66	97.43	**84.08**	91.67	77.24	81.4	72.22	86.18	84.75

**Table 10:** F-score (%) on selected categories from the Netease corpus.

	Model	Auto	Economy	Medicine	Military	Sports	Culture
**CBOW Based**	**CBOW**	95.15	90.08	91.19	93.92	90.76	97.51
	**CBOW+P**	95.54	90.7	91.56	**94.26**	91.39	97.68
	**CWE**	**95.65**	90.94	91.72	94.24	91.45	97.7
	**CWE+P1**	95.57	**91.05**	**91.75**	**94.26**	91.68	**97.74**
	**CWE+P2**	95.48	90.9	91.61	94.22	**91.69**	97.66
**Skip-gram Based**	**Skip-gram**	95.96	**91.65**	**92.24**	**94.78**	**92.13**	98.03
	**Skip-gram+P**	95.89	91.31	91.96	94.62	91.9	**98.15**
	**CWE**	96.09	91.46	92.06	94.59	91.94	98.09
	**CWE+P1**	**96.1**	91.35	92.23	94.6	91.85	98.11
	**CWE+P2**	95.92	91.54	92.09	94.72	92.03	98.13

**Table 11:** F-score (%) on selected categories from the TASS 2017 corpus.

	Model	Positive	Negative
**CBOW Based**	**CBOW**	**58.56**	73.86
	**CBOW+P**	57.84	**75.20**
**Skip-Gram Based**	**Skip-Gram**	**60.06**	**73.18**
	**Skip-Gram+p**	58.89	72.69

**Table 12:** F-score (%) on selected categories from the 20Newsgroups corpus.

	Model	comp	misc	politics	rec	religion	sci
**CBOW Based**	**CBOW**	76.93	**50.83**	69.31	80.43	73.83	61.19
	**CBOW+P**	**77.51**	50.38	**69.79**	**81.05**	**74.44**	**62.27**
**Skip-Gram Based**	**Skip-Gram**	80.33	**53.81**	**74.21**	83.42	**76.27**	**68.33**
	**Skip-Gram+P**	**81.24**	53.65	74.18	**83.87**	**76.27**	69.31

Several observations can be made from the preceding Tables. (1) In almost all languages and the corpus, the optimal accuracy is obtained by adding pronunciation information to the model. (2) Regardless of languages and the corpus, the optimal F-score in each category is generally obtained by a model that adds pronunciation information. (3) After adding the pronunciation embedding, the accuracy and F-score of each category are generally better than the benchmarks. For Chinese, the PWE based on word2vec generally outperforms word2vec, and the PWE based on the CWE is also generally better than the CWE. For English, the accuracy and the F-score of the PWE based on word2vec are generally better than that of word2vec on 20-Newsgroups. For Spanish, the accuracy and the F-score of “Negative” of the CBOW+P model is better than CBOW. The above 3 points demonstrate that including pronunciation information improves the performance of the word embedding model for different languages.

### Qualitative analysis

This section evaluates the quality of word embedding and pronunciation embedding by finding the words most similar to a given sound and the sounds most similar to a given word in the Chinese language. All embeddings were trained by CBOW+P. Cosine similarity is used to find the 4 most similar embeddings. The experimental results are shown in Tables 13 and 14.

**Table 13:** 4 most similar pinyin of the target word in diminishing order of similarity from Pinyin 1 to Pinyin 4.

Target word	Pinyin 1	Pinyin 2	Pinyin 3	Pinyin 4
**投降 (surrender)**	che4 (withdraw)	tui4 (withdraw)	jun1 (army)	bai4 (fail)
**财富 (wealth)**	cai2 (wealth)	chan3 (property)	zhai4 (debt)	huo4 (goods)
**体育 (sports)**	qiu2 (ball)	ping1 (ping-pong)	tiao4 (jump)	pang1 (ping-pong)

**Table 14:** 4 most similar words of the target word in diminishing order of similarity from Word 1 to Word 4.

Target pinyin	Word 1	Word 2	Word 3	Word 4
**bai4**	拝 (bow)	拜拜 (bye)	回拜(pay a return visit)	颓败 (decadent)
**shui4**	税负 (tax burden)	税源 (tax source)	睡意 (sleepiness)	睡袋 (sleeping bag)
**cai2**	横财 (windfall)	适才 (just now)	智财 (intelligent property)	财势 (fortune)

Table 13 shows that a semantic correlation exists between words and the pinyin that are the most similar to the word. For example, the 4 most similar pinyin of the word “投降” (surrender) are “che4”, “tui4”, “jun1” and “bai4”, where “che4” and “tui4” mean “撤退” (withdraw), “jun1” means “军队” (army) and “bai4” means “败” (fail). The words “财富” (wealth) and “体育” (sports) also have semantic correlations to similar pinyin.

According to Table 14, the words that are most similar to the pinyin contain the characters of those pinyin. For example, the 4 most similar words of pinyin “bai4” include the characters “败” (fail), “拝” (bow), and 和“拜” (bow), whose pinyin are “bai4”. The 4 most similar words of the pinyin “shui4” include the characters “睡” (sleep) and “税” (tax), whose pinyin are both “shui4”. The 4 most similar words of the pinyin “cai2” include the characters “才” (just) and “财” (wealth), whose pinyin are both “cai2”. This result demonstrates that the word embedding obtained by the PWE contains rich sound information.

## Conclusions

According to linguistic principles, spoken language is a direct expression of semantics, and written language is a record of spoken language. This paper proposes the PWE, which integrates word pronunciation into the word embedding model. The PWE is highly expandable from two aspects. First, the PWE can easily be combined with existing models such as word2vec and the CWE. Second, language is a storehouse of sound-images; therefore, the PWE can be applied to most languages. This paper introduces a variety of PWEs based on different existing models for different languages. Word similarity and text classification experiments demonstrate that the quality of word embedding is improved after adding sound information, which is beneficial to training. In addition, a qualitative embedding analysis revealed that word embedding contains rich sound information and that pronunciation embedding also contains semantic information. However, this paper adds pronunciation embedding to word embedding. The use of sound information is relatively simple and should be further utilized.
